# RORγt and RORα signature genes in human Th17 cells

**DOI:** 10.1371/journal.pone.0181868

**Published:** 2017-08-01

**Authors:** Glenda Castro, Xuejun Liu, Karen Ngo, Aimee De Leon-Tabaldo, Shanrong Zhao, Rosa Luna-Roman, Jingxue Yu, Tinghua Cao, Robert Kuhn, Patrick Wilkinson, Krystal Herman, Marina I. Nelen, Jonathan Blevitt, Xiaohua Xue, Anne Fourie, Wai-Ping Fung-Leung

**Affiliations:** Janssen R&D LLC, San Diego, California, United States of America; Seoul National University College of Pharmacy, REPUBLIC OF KOREA

## Abstract

RORγt and RORα are transcription factors of the RAR-related orphan nuclear receptor (ROR) family. They are expressed in Th17 cells and have been suggested to play a role in Th17 differentiation. Although RORγt signature genes have been characterized in mouse Th17 cells, detailed information on its transcriptional control in human Th17 cells is limited and even less is known about RORα signature genes which have not been reported in either human or mouse T cells. In this study, global gene expression of human CD4 T cells activated under Th17 skewing conditions was profiled by RNA sequencing. RORγt and RORα signature genes were identified in these Th17 cells treated with specific siRNAs to knock down RORγt or RORα expression. We have generated selective small molecule RORγt modulators and they were also utilized as pharmacological tools in RORγt signature gene identification. Our results showed that RORγt controlled the expression of a very selective number of genes in Th17 cells and most of them were regulated by RORα as well albeit a weaker influence. Key Th17 genes including IL-17A, IL-17F, IL-23R, CCL20 and CCR6 were shown to be regulated by both RORγt and RORα. Our results demonstrated an overlapping role of RORγt and RORα in human Th17 cell differentiation through regulation of a defined common set of Th17 genes. RORγt as a drug target for treatment of Th17 mediated autoimmune diseases such as psoriasis has been demonstrated recently in clinical trials. Our results suggest that RORα could be involved in same disease mechanisms and gene signatures identified in this report could be valuable biomarkers for tracking the pharmacodynamic effects of compounds that modulate RORγt or RORα activities in patients.

## Introduction

RORγt and RORα are transcription factors belonging to the RAR-related orphan nuclear receptor (ROR) family [1]. Proteins of the ROR family typically consist of 4 functional domains: an N-terminal (A/B) domain, a conserved DNA binding domain (DBD), a hinge domain, and a C-terminal ligand binding domain (LBD) [1]. RORγ and RORγt are two isoforms that are transcribed from the RORC gene, and four isoforms, RORα1–4, are produced from the RORA gene. These isoforms are generated from their corresponding genes through alternative promoter usage and exon splicing [2–5]. They differ in tissue expression profile and in the amino-terminal A/B domain that is critical for binding to specific ROR binding elements (RORE) to regulate target gene expression [1].

RORγt is expressed in distinct immune cell types including thymocytes, Th17 and Tc17 cells, γδ T cells, ILC3 cells, lymphoid tissue inducer (LTi) cells and NKp46^+^ CD3^-^ NK cells [6–9]. It is induced in activated CD4 T cells under Th17 differentiation conditions, such as the presence of IL-1β, IL-6, IL-23 and TGFβ [10–13]. The other isoform RORγ is known to be expressed in the liver, adipose, skeletal muscle and kidney, however its expression in T cells is still controversial [14–17]. RORα is present in a variety of tissues including liver, adipose, kidney, testis and the brain [2,3]. In the immune system, RORα is expressed in both lymphoid and myeloid cells and is induced during Th17 differentiation [18–20].

ROR transcription factors can work as repressors or activators on target gene transcription depending upon their recruitment of co-repressors or co-activators with their LBD domains, and this recruitment can be modulated by the type of ligands interacting with LBD [1]. Melatonin, cholesterol and cholesterol sulfate have been reported to be RORα ligands [20–23]. Sterol lipids, such as oxysterols, were recently identified as natural ligands for RORγt [24,25]. Small molecule modulators of RORγ and RORγt have been discovered and they bind to the LBD domain that is common for RORγ and RORγt and affect their recruitment of co-activators or co-repressors [26,27].

RORγt has been suggested to be important for Th17 differentiation by regulating the expression of Th17 genes [11]. RORγt signature genes in mouse T cells have been identified by global transcriptomic analysis of mouse T cells that were either defective in RORγt expression or activities as a result of gene targeting or selective inverse agonist treatment respectively [28,29]. The most pronounced effect was decreased expression of Th17 signature genes such as IL-17A, IL-17F, IL-22 and IL-23R, and increased expression of other T cell subset genes such as IL-4 and Tbx21. In human T cells, RORγt signature genes were reported in one study on memory CD4 T cells treated with a RORγt specific inverse agonist and gene expression profile was performed with gene microarray approach [30]. Further studies in human T cells with other approaches such as siRNA knock down of target genes, global gene expression profile with more sensitive platforms such as RNA-Seq, as well as using multiple RORγt compounds to exclude compound non-specific effects are warranted to confirm the findings and to explore the possible species difference between human and mouse in RORγt function.

RORα also contributes to Th17 differentiation, and RORα deficiency in mouse T cells has been reported to reduce expression of IL-17A, IL-23R, but not IL-17F or IL-22 [31]. Mouse T cells lacking expression of both RORγt and RORα showed a complete blockade in Th17 cell differentiation in contrast to the partial defects in T cells with a deficiency of either one, suggesting a complementary role for RORγt and RORα in Th17 commitment [31]. Global gene profiling to search for RORα gene signatures has not yet been conducted and information on the overlapping or unique signature genes between RORα and RORγt in Th17 cells is still pending for further investigation.

In this report, we used multiple RORγt and RORα selective siRNAs and RORγt inverse agonists to treat human CD4 T cells during Th17 differentiation, and performed RNA-Seq analysis to identify RORγt and RORα signature genes. Overlapping signature genes from RORγt and RORα were identified, and pathway analysis was performed to understand the functional roles of RORγt and RORα in the Th17 differentiation program.

## Materials and methods

### Materials

Four selective and potent RORγt inverse agonists referred to as compound A [32], B, C and D were generated and used as tools in this report.

Human blood samples were provided by the Scripps Research Institute. Blood donors have given consent to participate in this study and the study protocol on human blood samples was submitted by Janssen R&D and approved by the Scripps Research Institute IRB (Institutional Review Board).

Three small interfering RNAs (siRNAs) targeting RORγt were used in this study. Two pre-designed RORγt specific siRNAs (Stealth siRNA HSS109301, sequence ACAAATTGAAGTGATCCCTTGCAAA, HSS109302, sequence GCTGAGGGCAGAGAGAGCTTCTATA, and HSS109300, GGAGCAATGGAAGTGGTGCTGGTTA) named as RORC siRNA_1, RORC siRNA_2 and RORC siRNA_4 respectively were purchased from Invitrogen, USA, and one RORγt siRNA (Silencer Select s12111, sequence UGCUCUAUCUCUGUCAGGGAGTG) named as RORC siRNA_3 were purchased from Ambion, USA. In addition, two pre-designed siRNAs directed against RORα (Silencer Select s12103, sequence AUGCGAUUUAGAUAUAUUCTG, and s224549, sequence UGUAGUCACAUAUUGGUUCTG) labelled as RORA siRNA_1 and RORA siRNA_2 respectively, and three non-targeting siRNA (Stealth negative control medium GC duplex 46–2001 and Silencer negative control #1, AM4611 purchased from Amion, and negative siRNA AF488 from Allstars) labelled as control_1, control_2 and control_3 respectively.

Recombinant human RORγ, RORα and RORβ LBD proteins were prepared for ThermoFlour binding assay. The nucleotide sequences encoding the ligand binding domains (LBD) of human RORγ (Genbank accession no. NP005051, amino acid 237–497), RORα (Genbank accession no. NP599022, amino acid 304–556), and RORβ (Genbank accession no. NP008845, amino acid 201–452) were PCR-amplified and cloned into pET24a (Novagen) for expression in bacteria. All recombinant proteins contained an additional amino acid sequence of MAHHHHHHAGGAENLYFQGAMD at the N-terminal of LBD. After transformation of the bacteria strain BL21DE3 GOLD (Agilent) with the plasmids, 10 to 20 L cultures were grown to 1 OD and recombinant protein expression was induced overnight at 16°C with 0.2mM isopropyl-beta-D-thiogalactopyranoside (IPTG) (Corning). Bacteria cultures were lysed with microfluidizer in lysis buffer (20mM HEPES, pH 7.8, 500mM NaCl, 20mM imidazole, 2mM TECP). His-tagged proteins were collected with Ni-NTA resin (Qiagen) and purified with a HiTrap Q HP column (GE Life Sciences) eluted with a 40 CV gradient of 50-500mM NaCl.

### ThermoFluor^®^ binding assay

The ThermoFluor^®^ assay was established to measure the change in protein stability upon binding with compounds. 1-Anilinonaphthalene-8-Sulfonic Acid (1,8-ANS) (Invitrogen) was used as a fluorescent dye to label the test proteins. The assay was performed in polypropylene PCR microplates (TF-0384/k, Abgene) with a final assay of 3 μL reaction mixture per well overlaid with 1 μL silicone oil (Sigma-Aldrich). Assay plates were robotically loaded onto a thermostatically controlled PCR-type thermal block and then heated at a ramp-rate of 1°C/min. Fluorescence emission was initiated by continuous illumination with UV light (LC6, Hamamatsu) via fiber optic with a band-pass filter (380–400 nm) and detected with a CCD camera (Sensys, Roper Scientific) filtered to detect signal of 500 ± 25 nm. Reference wells contained recombinant LBD of RORγt, RORα or RORβ alone, and the assay conditions were as follows: 0.065 mg/mL RORγt LBD, 60 μM 1,8-ANS, 100 mM HEPES pH 7.0, 10 mM NaCl, 2.5 mM GSH, 0.002% Tween- 20; 0.1 mg/mL RORα, 70 μM 1,8-ANS, 25 mM HEPES pH 7.0, 50 mM NaCl, 0.001% Tween-20; 0.08 mg/mL RORβ, 60 μM 1,8-ANS, 50 mM HEPES pH 7.0, 100 mM NaCl, 0.001% Tween-20. All 3 assays were performed in the absence and in the presence of 100 μM co-activator peptide SRC1 (LTERHKILHRLLQEGSPSD, England Peptide custom synthesis). The binding affinity of test compounds was estimated as described previously [33].

### RORγt cellular reporter assay

This reporter assay was used to determine the agonistic or antagonist/inverse agonist activity of RORγt modulatory compounds on transcriptional activation driven by the RORγt LBD. HEK293T cells at 80% confluent were transfected with DNA constructs A and B using Fugene 6 (E2691, Invitrogen) at a 1:6 ratio of DNA and Fugene 6. DNA construct A is pBIND-RORγt LBD containing the wild type human RORγt LBD fused to the DNA binding domain of the GAL4 protein, and construct B is pGL4.31 (C935A, Promega) containing multiple GAL4 responsive DNA elements upstream of firefly luciferase. The pBIND vector contains the renilla luciferase gene under control of the SV40 promoter and Renilla luciferase expression is served as a control for transfection efficiency and cell viability. As a background control, cells were similarly co-transfected with an AF2 mutated RORγt LBD construct (LYKELF to LFKELF, pBIND-RORγt-AF2). The AF2 mutation has been shown to prevent co-activator binding to the RORγt LBD, thus preventing transcription of firefly luciferase.

Transiently transfected cells were plated into 96-well plates at 50,000 cells/well in phenol-red free DMEM, containing 5% lipid reduced FCS and Pen/Strep and treated with compounds for 24 hours. Media was removed and cells were lysed with 50 μL 1x Glo Lysis Buffer (Promega). Dual Glo Luciferase Reagent (50 μL/well) was then added and firefly luciferase luminescence was read on an Envision multilabel plate reader, after ten minutes incubation. Finally, Stop and Glo reagent (50 μL/well) was added and renilla luciferase luminescence was read on an Envision after ten minutes incubation. To calculate the effect of compounds on RORγt activity, the ratio of firefly to renilla luciferase was determined and plotted against compound concentration. Agonist compounds increase RORγt-driven luciferase expression, and antagonist or inverse agonist compounds decrease luciferase expression. IC_50_ values were determined using Graphpad Prism.

### Human CD4 T cell purification

Human PBMC were purified from two healthy donor blood using Ficoll-Paque (GE Healthcare) in density centrifugation. CD4 T cells were purified from human PBMC by negative selection with human CD4 T cell isolation kit (Miltenyi Biotec). After treatment with antibodies and magnetic beads, CD4 T cells were purified with SuperMacs using XS column (Miltenyi Biotec) and grown in culture medium. T cell culture medium was composed of IMDM, 2 mM L-glutamine, 1X NEAA, 1 mM sodium pyruvate, 100 U/ml penicillin, 100 μg/ml streptomycin, plus 10% KnockOut Serum Replacement (all from ThermoFisher Scientific).

### Compound IC_50_ determination in Th17 and Th1 differentiation assays

Effects of RORγt inverse agonists on human Th17 or Th1 differentiation was determined in T cell assays. CD4 T cells at 1.5 x 10^5^ cells per well in 96-well plate were pre-treated with compounds for 1 hour before activation with anti-CD3/anti-CD28 coated beads (Miltenyi Biotech) at 2:1 bead to cell ratio in RPMI-1640 culture medium containing 10% FCS, 2 mM glutamine, 1mM sodium pyruvate, 10mM HEPES, 1mM MEM nonessential amino acid solution, and 100 U/ml each of penicillin G and streptomycin (all culture reagents from Life Technologies). Th17 differentiation was initiated by supplementing culture medium with 20 U/ml IL-2, 10 ng/ml IL-1β (R&D), 10 ng/ml IL-23 (R&D), 50 ng/ml IL-6, 3 ng/ml TGFβ1 (R&D), 10 μg/ml of anti-IL-4 and anti-IFNγ (eBioscience). Th1 differentiation was driven by supplementing culture medium with 20 U/ml IL-2, 10 ng/ml IL-12 and 10 μg/ml anti-IL4 (eBioscience). After 3 days of incubation, culture supernatants were collected and the accumulated IL-17A in Th17 cultures and IFNγ in Th1 cultures were measured using MSD MULTI-SPOT^®^ Cytokine Plate following manufacturer’s instructions (Meso Scale Discovery). The plate was read using Sector Imager 6000, and IL-17A or IFNγ concentrations in samples were calculated from the standard curve. Proliferation of T cells in day 3 cultures was measured by 5 hours pulsing with 1μCi/well of 3H-thymidine (Perkin Elmer). Cells with incorporated radioactive thymidine were harvested onto glass fiber filter plates (Perkin Elmer). Filter plates were soaked with scintillant (Perkin Elmer) and radioactivity was counted using a Topcount (Packard). Compound IC_50_ values in proliferation and cytokine production were determined using GraphPad Prism.

### Compound or siRNA treatment of T cells in Th17 assay for gene expression study

Human CD4 T cells from 2 healthy donors were used in studies. 5.0 × 10^6^ human CD4 T cells were used per sample and cell samples were collected at 0, 24 and 48 hours of Th17 differentiation for gene expression studies with RNA-Seq and RT-PCR.

For compound treatment, CD4 T cells were pre-treated with compounds or DMSO vehicle control at 37°C for 1 hour before T cell activation. RORγt inverse agonists compound A, B and C were tested at two concentrations and DMSO treatments were used as controls in human Th17 differentiation assays as shown in the study plan (S1 Table).

For siRNA treatment, three RORγt siRNAs, two RORα siRNAs, two control siRNAs were tested at 1 μM, and mock transfected samples were included as controls (S1 Table). siRNA treatment was performed by Amaxa electroporation of human CD4 T cells with siRNAs. Human CD4 T cells were taken up in 100 μL Nucleofector solution (P3 primary cell 4D Nucleofector Kit, reference V4XP-3024, Amaxa Biosciences) at 5.0 × 10^6^ cells/100 μL. Cell suspensions were transfected with siRNA by electroporation using the FI-115 program in the Amaxa 4D-Nucleofector. T cells after electroporation were rinsed with 500 μL culture medium, transferred to 24-well culture plates and kept at 37°C cell incubator before use in T cell assays. It was noticed that Amaxa electroporation caused an increased expression of some genes in human T cells and this elevated gene expression lasted up to 8 hours after electroporation. To avoid gene expression study interfered by electroporation, human CD4 T cells were rested in culture medium at 37°C for 8 hours after siRNA electroporation before the use of these cells to initiate Th17 differentiation for transcriptome profile.

T cells after compound or siRNA treatments were stimulated with anti-CD3/anti-CD28 coated beads (Miltenyi Biotech) at 2:1 bead to cell ratio in culture medium containing 10 ng/ml IL1β (R&D), 10 ng/ml IL23 (R&D), 30 ng/ml TGFβ1 (R&D), 10 μg/ml of anti-IL4 and anti-IFNγ (eBioscience). T cell cultures were collected at different time points for RNA extraction and gene expression studies.

### RNA extraction

Total RNA was extracted from CD4 T cells using the RNeasy mini kit (Qiagen) following the manufacture’s instruction. CD4 T cells were lysed in RLT solution supplemented with β-mercaptoethanol and cell lysates were loaded in RNeasy mini spin column. DNA was removed by on-column DNase digestion and RNA was eluted from column with 50 μl nuclease-free water and stored at –80°C until analysis. RNA integrity was assessed with LabChip GX (PerkinElmer, USA).

### RNA-Seq

RNA-Seq based transcriptome profiling was conducted by Beijing Genomics Institute (Hong Kong) using the Illumina HiSeq^TM^ 2000 platform. The detailed procedures of RNA-Seq have been described previously [17]. In brief, RNA sequencing was performed for each sample to obtain about 40 million QC clean reads that were pair-end sequenced and with an average insert size of 160 base pairs and a typical read-length of 90 base pairs. Raw reads were mapped to the human reference genome (Reference Human.B37.3) and the expression of individual gene was quantified as the count of the mapped reads using RefGene20121217 as the gene model. The read alignment and gene expression quantification were conducted using Array Studio (www.omicsoft.com). Expression profiles of 25011 genes were generated for a total of 178 samples representing the independent cell cultures from the two donors under various treatments (S1 Table).

### Quantitative RT-PCR of RORα and RORγt signature genes

Expression of RORα, RORγt and IL-17A genes in siRNA or compound treated human Th17 cells was measured by quantitative RT-PCR using the TaqMan RNA-to-Ct 1 step kit (Applied Biosystems) following the manufacturer’s instructions. Validated TaqMan PCR primers and probes for detection of human IL-17A (Hs00936345_m1), RORα (Hs00536545_m1) and GAPDH (Hs02758991_g1), and custom made primers and probe for detection of RORγt (forward primer TGGACCACCCCCTGCTGAGAA GG, reverse primer CTTCAATTTGTGTTCTCATGACT, and probe GGGAGCCAAGGCCGG) were purchased from ThermoFisher. Real time PCR was performed using 600ng total RNA as template in the ABI PRISM 7500 Sequence Detection System (Applied Biosystems), with reaction starting at 48°C for 15 minutes, then 10 minutes at 95°C, followed by 40 cycles of 15 seconds at 95°C and 1 minute at 60°C.

RORγt signature genes in Th17 cells identified in RNA-Seq analysis were confirmed by quantitative RT-PCR. Duplicate samples from donor 1 treated with high concentrations of RORγt inverse agonists or DMSO were used in RTPCR confirmation. RNA samples were converted to cDNA using the high capacity cDNA reverse transcription kit (ThermoFisher) following the manufacturer’s instructions. TaqMan PCR primers and probes for target genes were purchased from ThermoFisher (S2 Table). Real time PCR was performed using cDNA samples as templates in the TaqMan array micro fluidic cards (Applied Biosystems) and ran in the QuantStudio 12K Flex System (Applied Biosystems). PCR reaction started at 50°C for 2 minutes, then 10 minutes at 95°C, followed by 40 cycles of 15 seconds at 95°C and 1 minute at 60°C.

PCR results were analyzed with the ddCT algorithm [34] to generate data on relative quantitation and changes in gene expression.

### Selection criteria on differentially expressed genes from RORα or RORγt siRNA treatment

For each of the two donors and at each of the two time points (24 and 48 hours) after Th17 activation, four comparisons (two RORα siRNAs against two scrambled siRNAs) and six comparisons (three RORγt siRNAs against two scramble siRNAs) were generated using general linear model to identify the differentially expressed genes in response to RORα or RORγt siRNA treatment respectively. In each comparison, differentially expressed genes were selected based on PV < 0.01 and fold change > 1.3. To further minimize the off-target effect associated with individual siRNA, genes that were differentially expressed in response to RORα siRNAs at each time point in each donor were selected on those that met the selection criteria in at least three of the all four comparisons; while genes that were differentially expressed in response to RORγt siRNA at each time point in each donor were selected on those that met the selection criteria in at least four of the six comparisons.

### Selection criteria on differentially expressed genes from treatments with RORγt inverse agonists

To analyze differential gene expression in response to RORγt compounds, 6 comparisons were generated using general linear model for each donor at each time point, including every compound at every concentration compared to the DMSO vehicle control. In each comparison, differentially expressed genes were selected based on false discovery rate (FDR) < 0.05 and fold change > 1.5. Genes that were differentially expressed in response to compound treatment at each time point were further selected on those fulfilling the selection criteria in both donors.

### IPA analysis

Upstream regulators and canonical pathways that were enriched by RORγt regulated genes were identified by Ingenuity Pathway Analysis [35].

## Result

### Characterization of RORγt inverse agonists used in gene signature study

**Fig 1:**
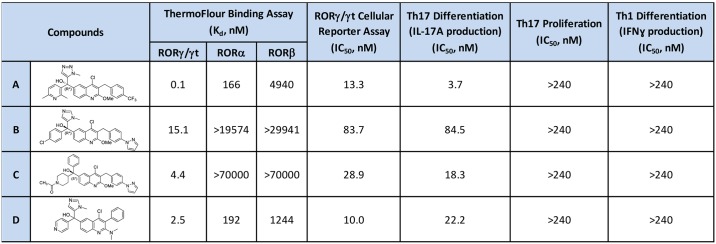
RORγt inverse agonists used in gene signature studies. Compound structures and their potencies in binding assay, cellular reporter assay, and T cell differentiation assay are shown. These compounds were selective in binding to RORγ/γt over RORα and RORβ, effective in blocking RORγt driven luciferase expression, inhibiting Th17 differentiation but not proliferation, and had no effect on Th1 differentiation.

RORγt compounds were identified from a high-throughput screen (HTS) of approximately 300,000 proprietary compounds using the ThermoFluor^®^ assay that measures binding to the human RORγt ligand binding domain (LBD) as a function of thermal stabilization [24]. Four RORγt inverse agonists referred to as compound A, B, C and D in this report were generated from optimization of quinolone tertiary alcohol HTS hits and they were used in gene signature studies [32]. These RORγt inverse agonists were shown to bind potently and selectively to RORγt LBD when compared to LBDs from other family members RORα and RORβ in the ThermoFluor^®^ assay (Fig 1). Evaluation of these compounds in a broader panel of 52 protein targets including receptors, ion channels and transporters confirmed the selectivity of these compounds which did not show more than 50% inhibition at 1 μM on any test targets (S3 Table). All these inverse agonists inhibited RORγt transcriptional activity in a reporter assay that measures RORγt driven luciferase expression (Fig 1). To validate their potency and efficacy in blocking RORγt mediated cellular response, compounds were tested in human Th17 and Th1 cell differentiation assays *in vitro* and shown to achieve significant inhibition of Th17 differentiation in IL-17A production (Fig 1). In contrast, these compounds were ineffective in blocking Th17 cell proliferation or IFNγ production in Th1 cell differentiation assay as described in Materials and Methods.

### Delayed effect of RORγt inverse agonists and siRNAs on IL-17A target gene expression

**Fig 2:**
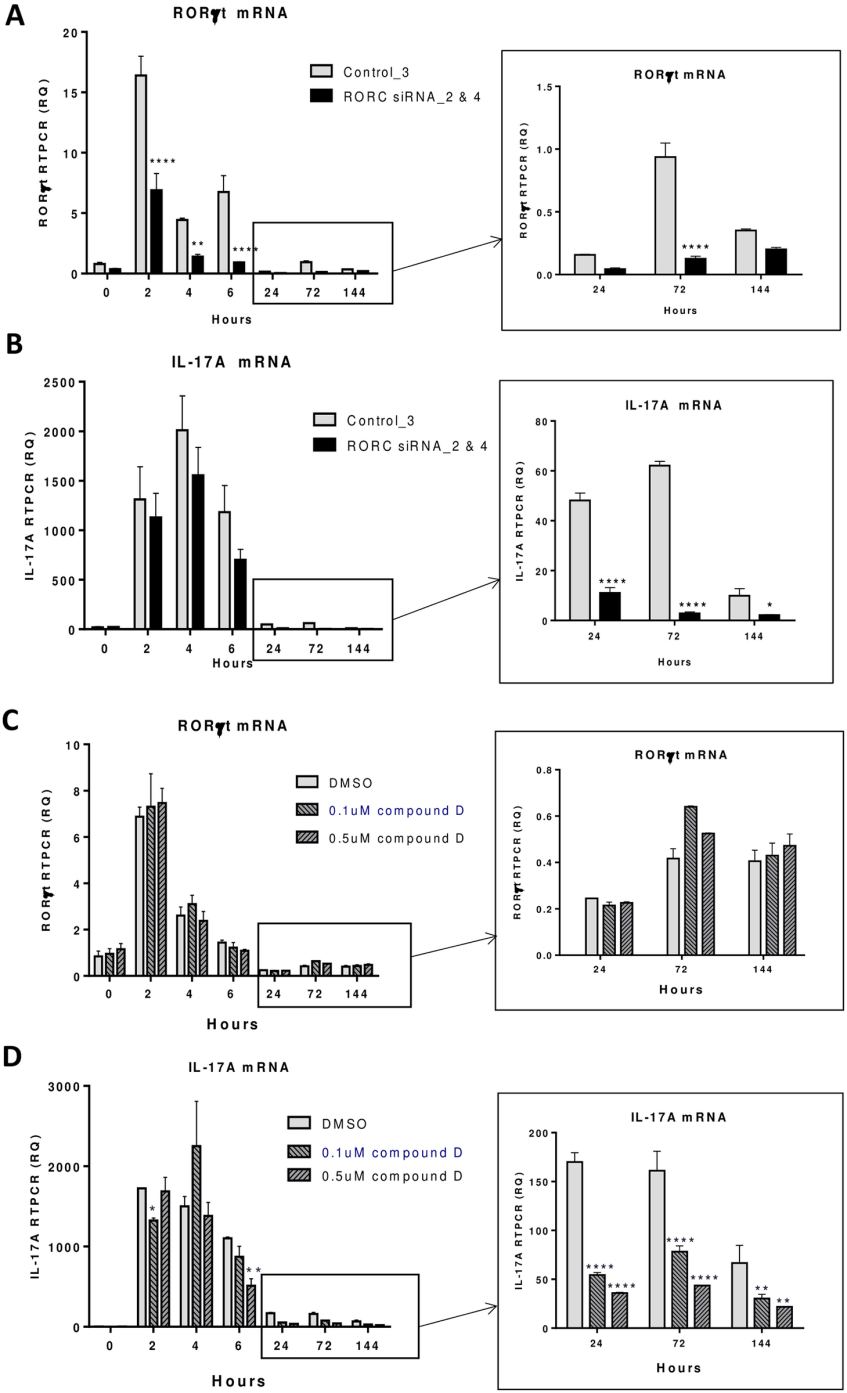
IL-17A transcript in human CD4 T cells reduced by RORγt siRNA or inverse agonist at later but not early stage of Th17 differentiation. Human CD4 T cells were activated under Th17 differentiation conditions as described in Material and Methods. RORγt siRNAs (RORC siRNA_2 & 4) and control scramble siRNA (control_3) were transfected into CD4 T cells prior to Th17 differentiation and samples were collected at different time points and the expression of RORγt (A) and IL-17A (B) transcripts was measured by RT-PCR as described in Materials and Methods. RORγt compound D at 0.1 and 0.5 μM and DMSO vehicle control were tested in a similar manner in Th17 differentiation. RORγt (C) and IL-17A (D) transcripts were measured by RT-PCR at different time points. Statistical significance of RORC siRNA or compound treated samples in reducing RORγt or IL-17A mRNA at different time points was analyzed with 2-way ANOVA and P values were indicated as *P<0.05, **P<0.01, ***P<0.001 and ****P<0.0001.

The effect on RORγt inverse agonists and siRNAs on human Th17 cell differentiation was tested in a time course study. RORγt specific siRNAs (RORC siRNA_2 & 4) and control scramble siRNA (control_3) as described in Methods and Materials were used to transfect human CD4 T cells before activation. RORγt and IL-17A transcripts were significantly induced in T cells at 2, 4 and 6 hour time points following anti-CD3/CD28 coated bead activation (Fig 2). In RORγt siRNAs transfected T cells, RORγt knock down was observed throughout the entire time course from 2 hours to 6 days, with a maximal effect of 86% at 6 and 72 hours, and 43% maintained on day 6 (Fig 2A). In contrast, levels of IL-17A transcript were not significantly reduced by RORγt siRNAs at early time points, with only 23% reduction at 4 hours after T cell activation (Fig 2B). However significant RORγt siRNA effects on IL-17A transcript were shown 1 day after T cell activation, with 77% and 95% reduced IL-17A levels at 24 and 72 hours, respectively, and 79% decrease maintained on day 6. We also explored the effect of RORγt inverse agonists in these studies. Unlike the siRNA knock down effects, RORγt compound D tested at 0.1 and 0.5 μM did not affect RORγt expression in T cells throughout the time course of the experiment (Fig 2C). However, a delayed effect on IL-17A transcripts similar to siRNA treated cells was observed. As shown in Fig 2D, compound D at 0.1 and 0.5 μM had similar effects on IL-17A transcripts and T cells with 0.5 μM compound D treatment resulted in 79% and 73% reduction of IL-17A transcripts at 24 and 72 hours, respectively, and 67% decrease maintained on day 6. Based on this finding, we decided to focus our search of RORγt signature genes at later time points, specifically on day 1 and day 2 of human Th17 differentiation.

### RORγt and RORα signature gene identification in human T cells

We took two different approaches to identify RORα and RORγt signature genes in human Th17 cells. We used RORγt inverse agonists to inhibit RORγt activity, and pursued the knock down approach using siRNAs specific for RORγt or RORα to remove their gene expression in human T cells. Human Th17 cells generated in the presence of compounds or siRNAs were then profiled in global gene expression using RNA-Seq to identify RORα and RORγt signature genes.

The study plan to identify RORγt and RORα signature genes in human T cells is as described in Materials and Methods (S1 Table). Three RORγt inverse agonists, namely compound A, B and C were tested at two concentrations that were higher than their IC_50_ values in human Th17 differentiation assays to ensure significant inhibition of RORγt transcriptional control on target genes (Fig 1). DMSO treated samples were used as controls to compare with compound treated samples. Three RORγt siRNAs, two RORα siRNAs, two control siRNAs, and mock transfected samples were also tested in same studies. Human CD4 T cells from 2 healthy donors were used and cell samples were collected for RT-PCR or RNA-Seq studies at 0, 24 and 48 hours of Th17 differentiation.

**Fig 3:**
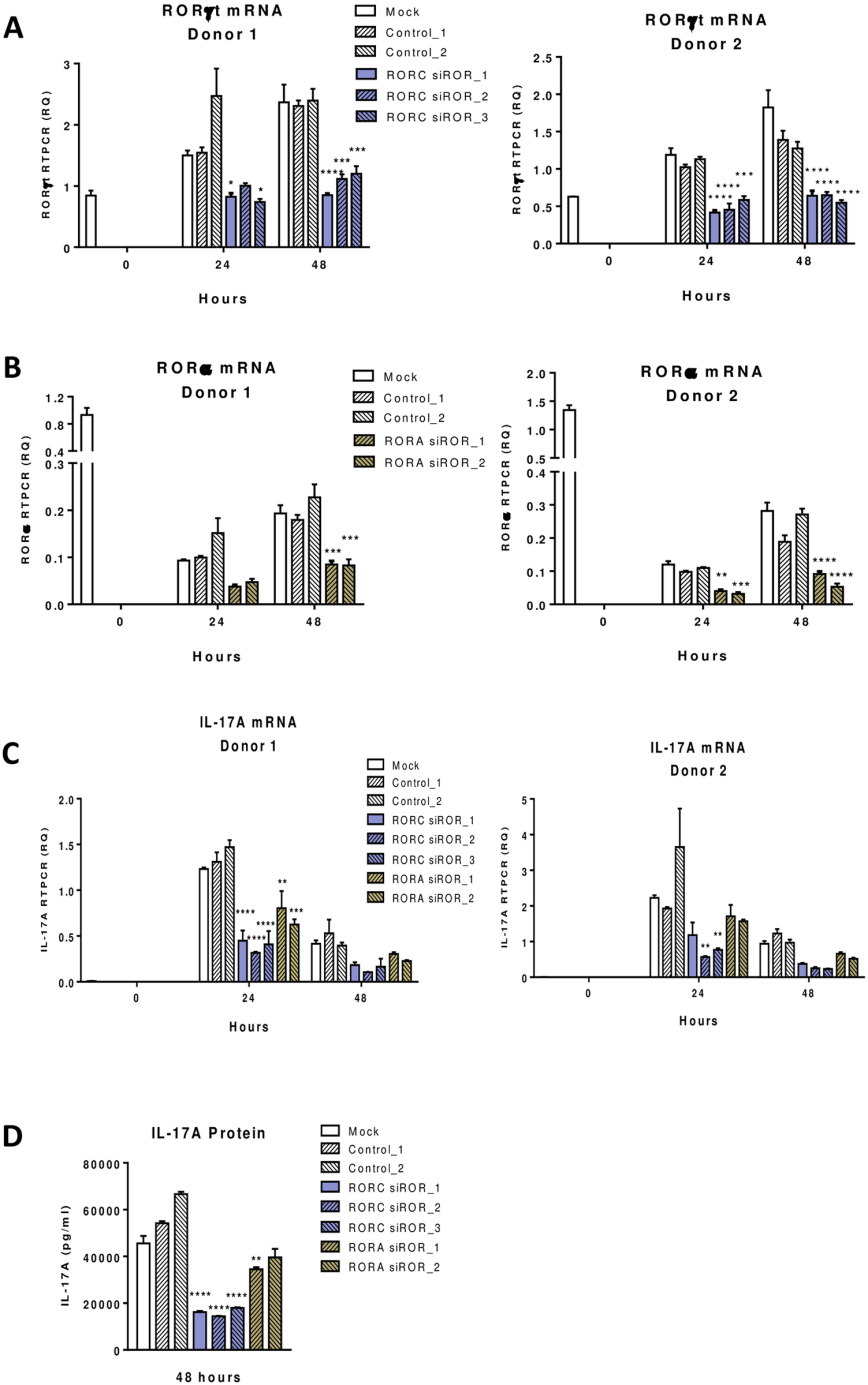
RORγt or RORα siRNA effect on IL-17A transcript in human CD4 T cells during Th17 differentiation. Human CD4 T cells purified from 2 donors were activated under Th17 differentiation condition as described in Material and Methods. RORγt and RORα siRNAs, as well as control siRNAs were transfected into CD4 T cells prior to Th17 differentiation and samples were collected at different time points as indicated. Expression of RORγt (A), RORα (B) and IL-17A (C) transcripts was measured by RT-PCR. IL-17A cytokine released in culture supernatants (D) was measured by ELISA. Statistical significance of the difference between RORC or RORA siRNA treated samples compared to mock samples at different time points was analyzed with 2-way ANOVA and P values were presented as *P<0.05, **P<0.01, ***P<0.001, and ****P<0.0001.

T cells transfected with RORγt siRNAs showed a significant knockdown of RORγt transcripts with 50% decrease at 24 hours, and up to 70% decrease at 48 hours (Fig 3A). RORα transcripts in T cells transfected with RORα siRNAs were reduced by 40% and 60% at 24 and 48 hours respectively (Fig 3B). The effect on IL-17A transcripts was 70–80% reduction by RORγt siRNAs and 40–50% by RORα siRNAs (Fig 3C). Measurement of IL-17A protein in supernatants of 2 day cultures showed a 70% and 50% reduction by RORγt and RORα siRNAs respectively (Fig 3D).

**Fig 4:**
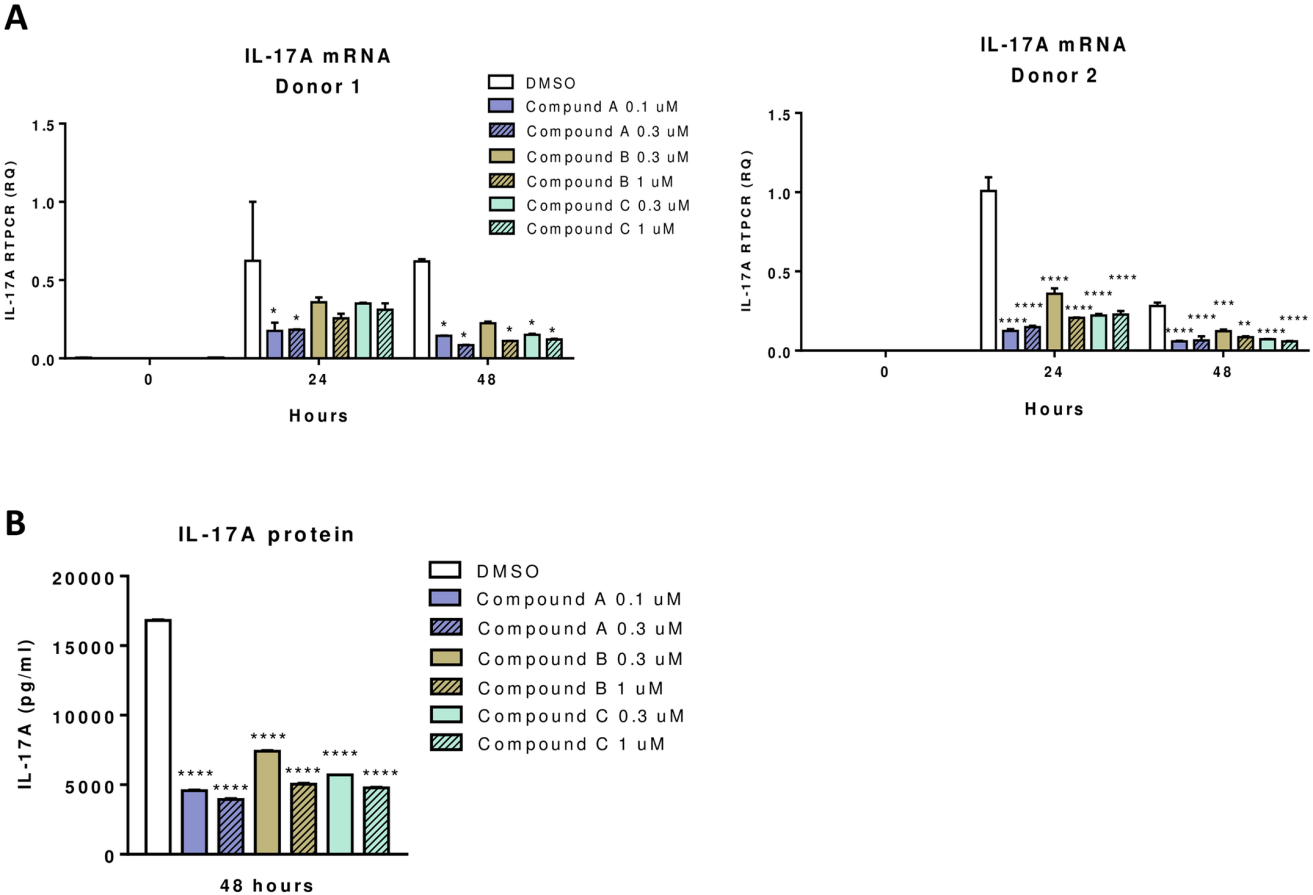
RORγt inverse agonist effect on IL-17A transcript in human CD4 T cells during Th17 differentiation. Human CD4 T cells purified from 2 donors were activated under Th17 differentiation condition as described in Material and Methods. CD4 T cells were treated with three RORγt inverse agonists at two different concentrations as well as DMSO vehicle control. Expression of IL-17A mRNA in different treated samples was measured by RT-PCR at time points as indicated (A). IL-17A cytokine released in culture supernatants (B) was measured by ELISA. Statistical significance of the difference between compound treated to DMSO samples at different time points was analyzed with 2-way ANOVA and P values were indicated as *P<0.05, **P<0.01, ***P<0.001 and ****P<0.0001.

IL-17A mRNA levels in T cells treated with RORγt inverse agonists A, B and C were detected by RT-PCR and compared to DMSO treated controls at different time points. Significant decrease in IL-17A transcripts was observed in all the inverse agonist treated samples with an average of 61% reduction at 24 hours and 83% at 48 hours (Fig 4A). IL-17A released in 2-day cell culture supernatants were reduced by an average of 69% in cell samples treated with inverse agonists (Fig 4B). In summary, expression of IL-17A in human Th17 cells was reduced by RORγt siRNAs or inverse agonists. RORα siRNA also decreased IL-17A expression but to an extent much less than that from RORγt siRNAs or inverse agonists.

The effects of RORγt and RORα siRNAs or inverse agonists on target gene IL-17A validated this study approach in signature gene identification. Global gene expression profile was conducted in all the T cell samples from siRNA and compound studies by RNA-Seq. RORγt and RORα gene signatures were identified through differential gene expression analysis as described in the following sections.

### RORγt and RORα4 isoforms expressed in human Th17 cells

**Fig 5:**
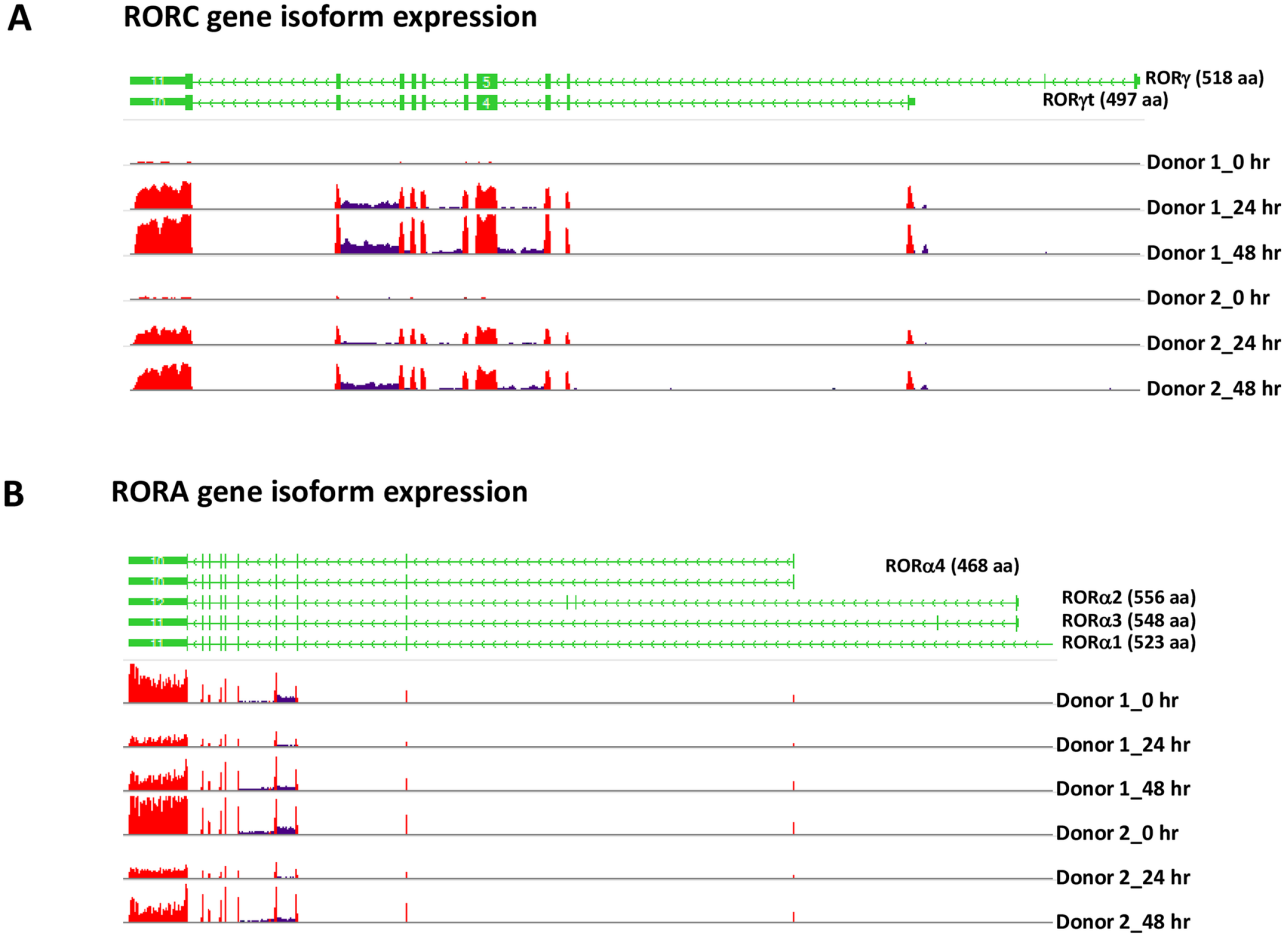
Expression of RORγt and RORα4 in human CD4 T cells during Th17 differentiation. Expression of RORγt isoform from the RORC gene (A) and RORα4 isoform from the RORA gene (B) was determined from detection of isoform specific exon sequences in RNA-Seq data analysis. Exons utilized by different isoforms of the RORC and RORA genes are shown in green, and the frequency of exon sequences in RNA-Seq data are shown in red in the histograms. DMSO treated human CD4 T cell samples from 2 donors at different time points of activation under Th17 differentiation conditions were used for isoform expression analysis.

Expression of specific isoforms of the RORA and RORC genes from alternative splicing was identified from RNA-Seq analysis of human T cell samples. As shown in Fig 5, RORC gene expression was minimal and below detection levels in CD4 T cells at the resting stage. Strong induction of RORC gene expression was detected in T cells at 24 and 48 hours after activation under Th17 polarizing conditions and only transcripts of the RORγt isoform were detected, as demonstrated with both donors (Fig 5A). In contrast, expression of the RORA gene was found in T cells at both resting and activated stages and only transcripts of the RORα4 isoform were detected, with expression levels higher in resting T cells and slightly reduced in activated T cells, as shown at 24 and 48 hours in both donors (Fig 5B).

### RORγt signature genes in human Th17 cells identified with siRNAs or inverse agonists

Three siRNAs specific for the RORC gene were used in signature gene studies in human CD4 T cells. Although the sequences of these siRNAs were in the common region utilized by the RORγ and RORγt splice variants, signature genes are expected to be from RORγt, since it was the only isoform detected in human T cells (Fig 5A).

**Fig 6:**
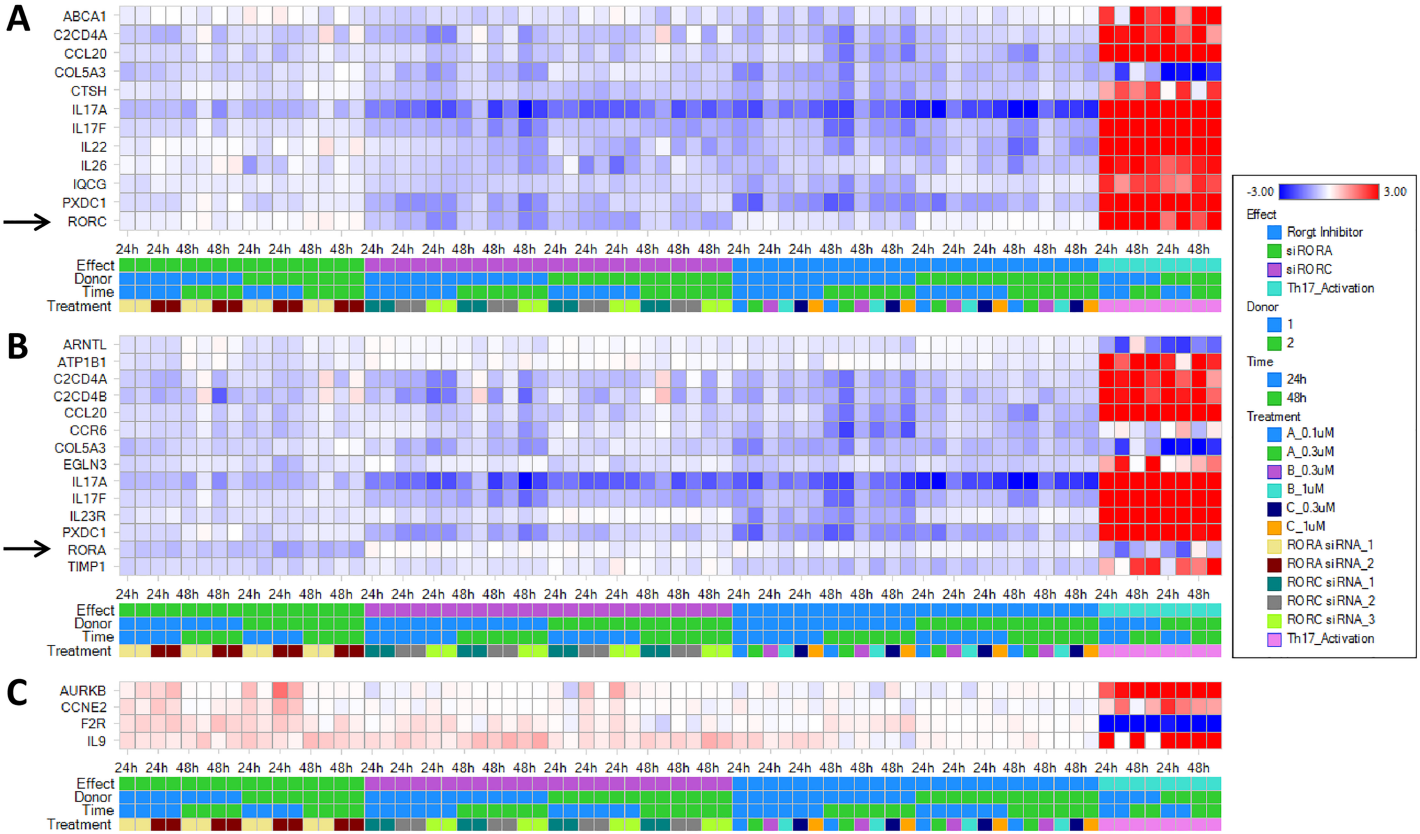
Global gene expression profile with RNA-Seq identified genes differentially regulated by RORγt or RORα siRNA in human CD4 T cells during Th17 differentiation. RNA sequencing profile was performed on RNA samples extracted from human CD4 T cells treated with RORγt or RORα siRNA, or RORγt inverse agonists as described in S1 Table. Genes down-regulated by RORγt (A) and RORα (B) siRNAs, or up-regulated by RORγt or RORα siRNAs (C) were identified in analysis of RNA-Seq data using the criteria described in Materials and Methods. Each column represents log2 ratios of selected genes in one of the 72 comparisons, including comparison of untreated Th17 cells with RORγt or RORα siRNA transfected cells or RORγt inverse agonist treated cells at two time points and T cells from 2 independent donors.

During the time course of Th17 differentiation, expression of 12 genes, including RORC, IL-17A, IL-17F, IL-22, IL-26, ABCA1, C2CD4A, CCL20, COL5A3, CTSH, IQCG, and PXDC1, were reduced by treatment of T cells with RORγt siRNAs (Fig 6A). All these genes except for COL5A3 were genes with induced expression in Th17 cells at 24 and 48 hours of differentiation. IL-9 was an induced gene in Th17 cells and interestingly its expression was enhanced by RORγt siRNAs (Fig 6C).

**Fig 7:**
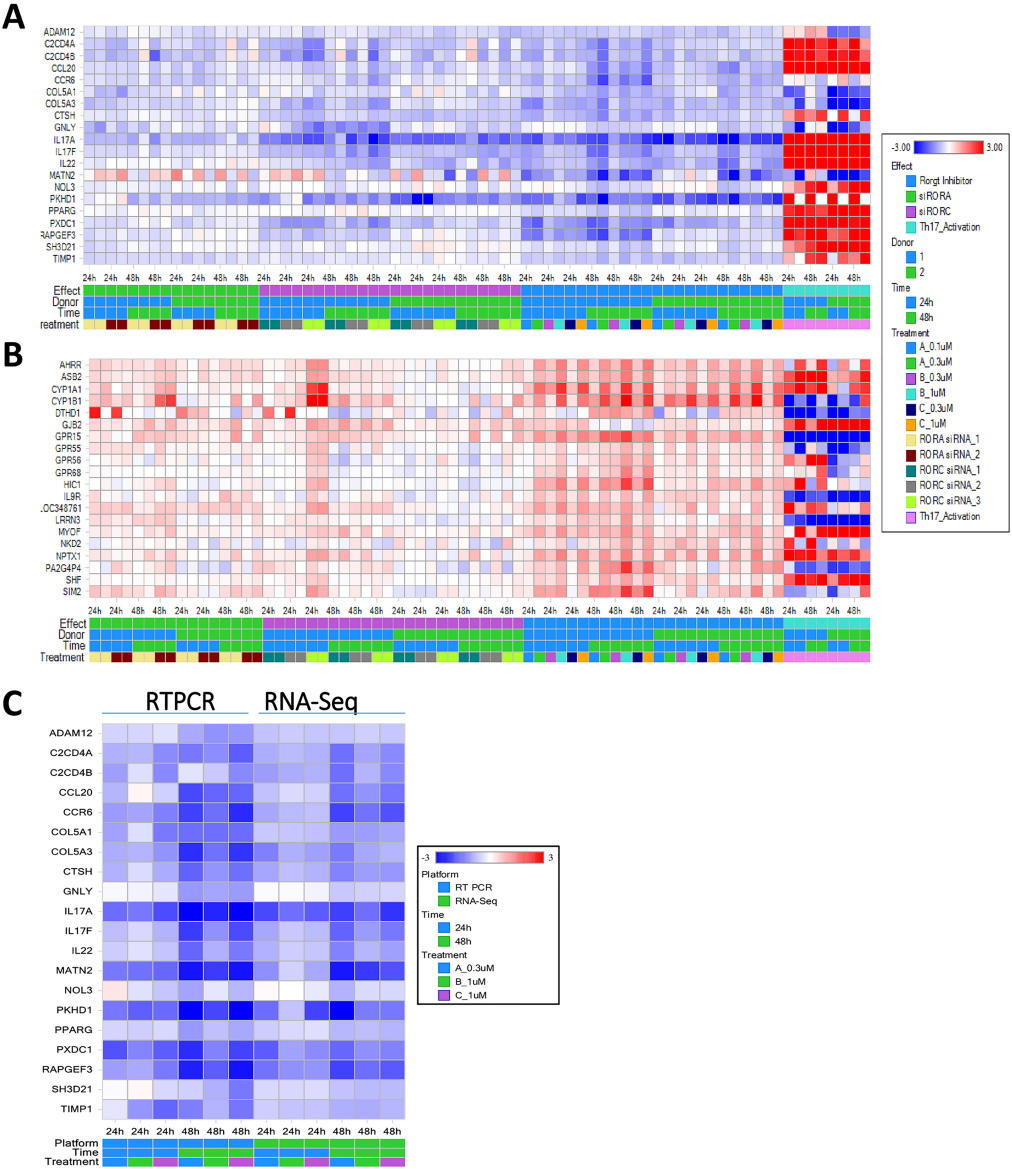
Global gene expression profile with RNA-Seq identified genes down- or up-regulated by RORγt inverse agonists in human CD4 T cells during Th17 differentiation. RNA sequencing profile was performed on RNA samples extracted from human CD4 T cells treated with 3 different RORγt inverse agonists at 2 concentrations as described in S1 Table. Genes down-regulated (A) or up-regulated (B) by RORγt inverse agonists were identified in analysis of RNA-Seq data using the criteria described in Materials and Methods. Each column represents log2 ratios of selected genes in one of the 72 comparisons, including comparison of untreated Th17 cells with RORγt or RORα siRNA transfected cells or RORγt inverse agonist treated cells at two time points and T cells from 2 independent donors. Genes down-regulated by RORγt inverse agonists in Th17 cells identified from RNA-Seq analysis were confirmed by RTPCR of donor 1 samples as described in Materials and Methods (C). Heat map of log2 ratios of gene expression in response to treatment of RORγt inverse agonists determined by RNA-Seq and RTPCR were compared.

Studies with RORγt inverse agonists identified 20 genes with expression reduced by any one of the three RORγt inverse agonists in both donor T cells (Fig 7A). Most of them except for MATN2 were also down-regulated by RORγt siRNAs, and 14 of them were induced genes in Th17 cells at 24 and 48 hours of differentiation. There were 20 genes with expression induced by any one of the three RORγt inverse agonists in both donor T cells in a dose-dependent fashion (Fig 7B). Among them 8 were induced genes and 4 were suppressed genes in activated Th17 cells at 24 and 48 hours of culture. The differential gene expression in response to these inverse agonists was more evident in donor 1 than in donor 2. Genes down-regulated by RORγt inverse agonists identified from RNA-Seq analysis were further confirmed by RTPCR on donor 1 samples (Fig 7C). In summary, RORγt signature genes were identified with two different approaches using RORγt specific siRNAs or inverse agonists.

### RORα4 signature genes in human Th17 cells identified with RORα siRNAs

Two siRNAs specific for the RORA gene were used to knock down expression of RORα transcripts in human CD4 T cells. Although the sequences of these siRNAs were in the region common for all splice variants of the RORA gene, signature genes identified in this study are expected to be from RORα4, since it was the only isoform detected in human T cells (Fig 5B).

Most of the RORα4 signature genes identified in this study were up-regulated in Th17 cells and reduced by RORα siRNA treatments. As shown in Fig 6B, expression of RORα4 and 13 additional genes were reduced by treatment of RORα siRNAs in both donor T cells. Among the 13 signature genes, 11 of them were induced genes in Th17 cells. There were 3 genes AURKB, CCNE2, and F2R moderately induced by RORα siRNAs in T cells from both donors (Fig 6C). AURKB and CCNE2 were induced genes in Th17 cells whereas F2R was a Th17 suppressed gene.

### RORα4 and RORγt common signature genes in human Th17 cells

Results from our study showed that RORα4 and RORγt have shared signature genes including IL-17A, IL-17F, C2CD4A, C2CD4B, CCL20, CCR6, COL5A3, PXDC1, and TIMP1 (Figs 6A, 6B and 7A). Although IL23R and EGLN3 were identified with RORα siRNAs, and CTSH, IL-26, ADAM12, COL5A1, GNLY, PKHD1, RAPGEF3, CYP1A1 and CYP1B1 were from RORγt signature search, careful review of these genes in expression heat maps revealed a similar trend of differential gene expression with approaches targeting RORα4 or RORγt (Figs 6 and 7), suggesting that they could be common signatures for RORγt and RORα4 as well. In general, the effect of RORα siRNAs on signature gene expression was less than RORγt siRNAs or inverse agonists. In summary, RORα4 and RORγt shared some of their signature genes in Th17 cells and RORγt had a more significant role than RORα4 in regulating signature gene expression.

### RORγt pathways and upstream regulators identified in Ingenuity Pathway Analysis

Most of the genes with expression down-regulated by RORγt siRNA overlapped with genes reduced by RORγt inverse agonists (Figs 6A & 7A). A total of 24 RORγt signature genes that were down-regulated by either siRNAs or compounds were generated from differential gene expression analysis (Table 1). These genes were used to conduct Ingenuity Pathway Analysis (IPA) to identify the corresponding upstream regulators (Table 2) and canonical pathways (Table 3). As shown in Table 2, the RORC and RORA genes, as well as the RORγt inverse agonist TMP778 were identified by IPA in the list of top 20 upstream regulators, validating our approach in RORγt and RORα signature gene identification, and confirming the overlapping functions of these two transcription factors. In addition, regulators and pathways involved in Th17 cell differentiation and IL-17 mediated effects were pulled out from IPA analysis (Tables 2 and 3). The results are consistent with other reports on the role of RORγt and RORα in modulating Th17 cell generation and functions [11,12,29,31].

**Table 1:** A total of 24 genes were down-regulated by RORγt siRNAs or inverse agonists in human Th17 cells. The change in gene expression resulted from treatment of RORγt siRNAs or RORγt inverse agonists was presented in fold change of the geomean average calculated from samples of the two donors.

Gene	Down-regulated by RORγt siRNAs	Down-regulated by RORγt inverse agonists	GeoMean Fold Change by RORγt siRNAs	GeoMean Fold Change by RORγt inverse agonists
ABCA1	Yes		-1.3	-1.3
ADAM12		Yes	-1.4	-1.5
C2CD4A	Yes	Yes	-1.6	-1.7
C2CD4B		Yes	-1.7	-1.8
CCL20	Yes	Yes	-1.4	-1.8
CCR6		Yes	-1.4	-1.9
COL5A1		Yes	-1.2	-1.7
COL5A3	Yes	Yes	-1.6	-1.9
CTSH	Yes	Yes	-1.6	-1.7
GNLY		Yes	-1.7	-1.3
IL17A	Yes	Yes	-3.4	-4.3
IL17F	Yes	Yes	-1.9	-2.0
IL22	Yes	Yes	-1.6	-2.0
IL26	Yes		-1.7	-1.6
IQCG	Yes		-1.5	-1.5
MATN2		Yes	-1.0	-2.5
NOL3		Yes	-1.1	-1.2
PKHD1		Yes	-2.8	-3.6
PPARG		Yes	-1.0	-1.3
PXDC1	Yes	Yes	-1.8	-2.3
RAPGEF3		Yes	-1.4	-1.9
RORC	Yes		-1.9	-1.2
SH3D21		Yes	-1.1	-1.3
TIMP1		Yes	-1.2	-1.6

**Table 2:** Upstream regulators of the 24 genes down-regulated by RORγt siRNAs or inverse agonists in human Th17 cells were predicted from IPA analysis and the top 20 regulators are shown. These regulators were ranked by their P values which reflect the enrichment of the 24 genes regulated directly or indirectly by these upstream regulators. The activation status of upstream regulators was determined from the treatment effect on the involved genes and presented as activation z-scores, with positive value for activated status, and negative value for inhibited status.

Upstream Regulator	Activation Z-score	P-value of Overlap	Involved Genes
TMP778 (RORγt inverse agonist)	2.39	4.25E-18	CCL20,CCR6,IL17A,IL17F,IL22,IL26
IL23	-2.57	5.80E-15	CCL20,CCR6,IL17A,IL17F,IL22,IL26,RORC
IL23R	-1.98	3.03E-11	IL17A,IL17F,IL22,RORC
AMG 827 (anti-IL-17RA)	2.00	8.61E-10	CCL20,IL17A,IL17F,IL22
TGFB1	-2.38	1.18E-09	ABCA1,ADAM12,CCL20,CCR6,COL5A1,CTSH,IL17A,IL17F,IL22,PPARG,RAPGEF3,RORC,TIMP1
IL2	-2.14	2.24E-09	ABCA1,CCR6,IL17A,IL17F,IL22,IL26,PPARG,RORC,TIMP1
IL27	1.48	3.04E-09	CCL20,CCR6,IL17A,IL17F,IL22,RORC
NFATC2	1.00	6.99E-09	ABCA1,IL17A,IL17F,IL22,PPARG,RORC
RORC	-2.16	8.88E-09	CCL20,CCR6,IL17A,IL17F,IL22,RORC
Secretase gamma	-1.96	9.08E-09	IL17A,IL17F,IL22,RORC
prostaglandin E2	-1.10	1.40E-08	CCL20,CCR6,IL17A,IL17F,IL22,PPARG,RORC
ZBTB16	-2.21	1.49E-08	CCR6,IL17A,IL17F,IL22,RORC
IL6	-2.57	1.62E-08	ABCA1,CCL20,CCR6,IL17A,IL17F,IL22,PPARG,RORC,TIMP1
NFATC1	-2.17	1.81E-08	IL17A,IL17F,IL22,PPARG,RORC
PLP1	-2.00	2.68E-08	CCL20,CCR6,IL17A,TIMP1
AHR	0.48	2.72E-08	COL5A1,IL17A,IL17F,IL22,MATN2,PPARG,RORC
IL1	-1.56	3.22E-08	CCL20,CCR6,IL17A,IL22,PPARG,RORC,TIMP1
RORA	-2.40	3.24E-08	ABCA1,CCR6,IL17A,IL17F,IL22,RORC
IL23A	-1.99	3.48E-08	IL17A,IL17F,IL22,RORC
IL17A	-0.70	5.24E-08	CCL20,IL17A,IL17F,IL22,PPARG,TIMP1

**Table 3:** Top 10 canonical pathways in IPA associated with the 24 genes that were down-regulated by RORγt siRNAs or inverse agonists in human Th17 cells. The top 10 canonical pathways were selected by the p-value of overlap of the 24 genes and the genes in each pathway.

Ingenuity Canonical Pathways	-log(p-value)	Involved Genes
Role of Cytokines in Mediating Communication between Immune Cells	6.24	IL26,IL22,IL17F,IL17A
T Helper Cell Differentiation	4.03	RORC,IL17F,IL17A
IL-17 Signaling	4.01	TIMP1,IL17F,IL17A
Role of IL-17A in Psoriasis	3.94	CCL20,IL17A
Differential Regulation of Cytokine Production in Macrophages and T Helper Cells by IL-17A and IL-17F	3.65	IL17F,IL17A
Differential Regulation of Cytokine Production in Intestinal Epithelial Cells by IL-17A and IL-17F	3.43	IL17F,IL17A
IL-17A Signaling in Gastric Cells	3.36	CCL20,IL17A
Inhibition of Matrix Metalloproteases	2.97	ADAM12,TIMP1
Hepatic Fibrosis / Hepatic Stellate Cell Activation	2.83	COL5A1,COL5A3,TIMP1
Role of IL-17A in Arthritis	2.69	CCL20,IL17A

## Discussion

RORγt is a key transcription factor playing an important role in Th17 cell differentiation [11]. Although expression of RORγ has been reported in T cells [16], we only detected RORγt but not RORγ in our previous report on RNA-Seq analysis of human CCR6^+^ CD4 memory T cells [17]. RNA-Seq data from this study also confirmed our earlier finding that only RORγt but not RORγ was expressed in human Th17 cells. RORα2 and RORα4 are the two isoforms from the RORA gene and their expression in T cells have been reported previously [18]. RNA-Seq data from this study however only detected RORα4 but not the other isoforms from the RORA gene in human Th17 cells.

We conducted a time course study to track the effects of RORγt siRNAs or inverse agonists on human Th17 differentiation. RORγt target gene IL-17A was highly induced at early time points within the first day of T cell activation. However neither RORγt specific siRNAs nor inverse agonists had significant impacts on IL-17A gene expression on the first day of T cell activation. The effect on IL-17A gene expression was observed only on the second day after the peak level of IL-17A gene expression. The results suggest that RORγt may be more involved in sustaining the late phase expression of target genes than the initial phase of gene induction. The delayed effect of RORγt on target gene expression also highlights its function on Th17 cell differentiation which takes place later in the course of T cell activation.

Although RORγt signature genes have been characterized in mouse Th17 cells, information on human T cells from global transcriptional profile was reported only in one study [28–30]. RORα has been suggested to play a role in regulation of Th17 gene expression [18–20,31], however study of RORα signature genes from global gene profile has not been reported previously. To identify RORγt and RORα signature genes in human T cells, we conducted the global gene expression profile in human CD4 T cells under Th17 differentiation conditions after treatment with RORγt or RORα siRNAs, or small molecule RORγt inverse agonists. To build the confidence in signature gene data, multiple siRNAs and inverse agonists were used with test samples in replicates and experiments repeated with CD4 T cells from 2 healthy donors. Global gene expression was measured with RNA-Seq to ensure high detection sensitivity. Conservative analytical algorithms were utilized to determine signature genes for RORγt and RORα4, the two isoforms expressed in human Th17 cells. Using this approach we were able to identify RORα4 and RORγt signature genes in human Th17 cells. It is also the first report on RORα4 signature genes in Th17 cells from global transcription analysis.

The majority of RORα4 and RORγt signature genes were induced genes in Th17 cells and positively regulated by these transcription factors, as demonstrated by their expression being reduced by siRNA or inverse agonist treatments. Furthermore, expression of these signature genes was not completely blocked by siRNAs or inverse agonists. IL-17A was the most significantly affected gene amongst all signature genes, and yet the reduction of transcript and protein levels was not complete at maximal inhibition effects. The effect of RORα4 in regulation of target gene expression was more subtle than RORγt. Our study also showed an overlapping role of RORα4 and RORγt in regulating a small number of key Th17 genes including IL-17A, IL-17F, IL-22, CCL20 and CCR6. These genes have been reported to be important for Th17 differentiation and transcriptionally regulated by RORγt in human and mouse Th17 cells [28–30]. We demonstrated in this report that RORα4 also participated in the regulation of these genes in Th17 cells. In addition, other genes such as C2CD4A, PXDC1, TIMP1 and COL5A3 were found to be regulated by both RORα4 and RORγt in Th17 cells as well and the function of these genes remain unclear and warrant further investigations.

Th17 cells play a key role in the pathogenesis of many autoimmune diseases such as psoriasis, rheumatoid arthritis, inflammatory bowel disease and multiple sclerosis [36,37]. Antibodies targeting IL-17A and the IL-17 receptor IL-17RA have shown clinical efficacy in psoriasis, rheumatoid arthritis, and uveitis [38–40]. RORγt compound can selectively regulate Th17 signature gene expression in mononuclear cells isolated from both the blood and affected skin of psoriasis patients and inhibited IL-23 induced IL-17A from psoriasis patient PBMC [30]. Since RORγt and RORα play a significant role in Th17 differentiation and IL-17A production, identification of their target genes in Th17 cells may lead to discovery of candidate genes with potential value for new therapeutic approaches.

## Supporting information

S1 Table RORγt and RORα gene signature study plan. Numbers of cell culture samples with different treatments at multiple time points in gene expression analysis are shown. The various culture conditions are defined by pairs of treatments (rows) and time points (columns) after activation of CD4 T cells from two donors.
(DOCX)

S2 Table RORγt signature genes identified in RORγt compound treated Th17 cells were confirmed by RTPCR using TaqMan PCR primers and probes from ThermoFisher and their catalogue IDs are shown. (DOCX)

S3 Table Selectivity profile of RORγt inverse agonists A, B, C and D. Compounds were evaluated at 1 μM and 10 μM in a panel of assays against 50 receptors, ion channels and transporters (Profile service by Cerep Inc.).
(DOCX)
