# Supplementary material for: RORγt and RORα signature genes in human Th17 cells
Source: PLoS One. 2017 Aug 1;12(8):e0181868. doi: 10.1371/journal.pone.0181868 (PMC5538713; doi:10.1371/journal.pone.0181868)
Supplement: S1 Table — Numbers of cell culture samples with different treatments at multiple time points in gene expression analysis are shown. The various culture conditions are defined by pairs of treatments (rows) and time points (columns) after activation of CD4 T cells from two donors. (DOCX) [file pone.0181868.s001.docx]

**S1 Table**. **RORɣt and RORα gene signature study plan.** Numbers of cell culture samples with different treatments at multiple time points in gene expression analysis are shown. The various culture conditions are defined by pairs of treatments (rows) and time points (columns) after activation of CD4 T cells from two donors.
