# Supplementary material for: RORγt and RORα signature genes in human Th17 cells
Source: PLoS One. 2017 Aug 1;12(8):e0181868. doi: 10.1371/journal.pone.0181868 (PMC5538713; doi:10.1371/journal.pone.0181868)
Supplement: S2 Table — (DOCX) [file pone.0181868.s002.docx]

**S2 Table. RORɣt signature genes identified in RORɣt compound treated Th17 cells were confirmed by RTPCR using TaqMan PCR primers and probes from ThermoFisher and their catalogue IDs are shown.**
