# Supplementary material for: RORγt and RORα signature genes in human Th17 cells
Source: PLoS One. 2017 Aug 1;12(8):e0181868. doi: 10.1371/journal.pone.0181868 (PMC5538713; doi:10.1371/journal.pone.0181868)
Supplement: S3 Table — Compounds were evaluated at 1 μM and 10 μM in a panel of assays against 50 receptors, ion channels and transporters (Profile service by Cerep Inc.). (DOCX) [file pone.0181868.s003.docx]

**S3 Table. RORɣt inverse agonists A, B, C and D were evaluated at 1 µM and 10 µM in a panel of assays against 50 receptors, ion channels and transporters (Profile service by Cerep Inc.).**

| **Target** | **% Inhibition at 1µM** | | | | **% Inhibition at 10µM** | | | |
| --- | --- | --- | --- | --- | --- | --- | --- | --- |
|  | **A** | **B** | **C** | **D** | **A** | **B** | **C** | **D** |
| A1 (h) | 19 | - | - | 18 | 30 | - | - | 50 |
| A2A (h) | 20 | - | 18 | 2 | 5 | - | 21 | 43 |
| A3 (h) | 49 | 9 | 16 | - | 92 | 20 | 41 | 67 |
| Aa1 | 8 | - | - | - | 5 | - | - | 1 |
| Aa2 | - | - | - | - | 7 | - | - | 18 |
| Ab1 (h) | 0 | 0 | - | 3 | 10 | 2 | - | 2 |
| AT1 (h) | 0 | 1 | - | - | 32 | 16 | 11 | 28 |
| BZD | - | - | - | - | - | - | - | - |
| BK2 (h) | 6 | 9 | - | - | - | 6 | - | 10 |
| CCKA (h) | - | - | - | 11 | - | - | - | - |
| D1 (h) | 16 | 10 | - | - | 48 | 29 | 11 | 40 |
| D2S (h) | 12 | 7 | 10 | 7 | 70 | - | - | 25 |
| ETA (h) | 5 | - | - | - | 4 | - | 12 | - |
| GABA | 8 | 9 | - | - | - | - | 12 | 13 |
| GAL2 (h) | - | - | - | - | - | 1 | - | - |
| CXCR2 (h) | - | - | - | - | 12 | - | 10 | 17 |
| CCR1 (h) | - | - | 17 | - | - | - | - | 6 |
| DA-T | 38 | - | 17 | 23 | 81 | - | 25 | 87 |
| H1 (h) | - | - | 10 | - | 8 | 2 | - | 27 |
| H2 (h) | - | - | - | - | - | - | - | 5 |
| M1 (h) | - | - | 12 | 6 | - | - | 21 | 67 |
| M2 (h) | - | 16 | - | 10 | - | 23 | - | 47 |
| M3 (h) | - | 1 | 21 | 33 | 63 | 15 | 19 | 70 |
| MC4 (h) | - | - | 14 | 3 | 9 | 6 | - | 25 |
| ML1 | - | 13 | 4 | 5 | 39 | 38 | 27 | 37 |
| NE-T | 44 | 21 | 19 | 5 | 86 | 38 | 38 | 76 |
| NK2 (h) | 7 | 9 | - | 9 | 41 | 30 | 19 | 98 |
| NK3 (h) | 9 | 1 | 21 | - | 57 | 10 | 31 | 45 |
| NT1 (h) | - | - | - | - | - | - | - | - |
| OP1 (h) | 9 | 6 | 9 | - | 27 | 2 | 11 | 36 |
| OP2 | 9 | - | - | - | 37 | 10 | 12 | 23 |
| OP3 (h) | - | 3 | 2 | 3 | 17 | 3 | 15 | 30 |
| ORL1 (h) | 1 | 6 | - | 5 | 10 | 4 | - | - |
| 5-HT6 (h) | - | - | 20 | - | 10 | - | 22 | 18 |
| 5HT7 (h) | 4 | - | - | - | 4 | - | - | 16 |
| 5HT1A (h) | 14 | - | - | - | 19 | - | - | - |
| 5HT1B | - | - | - | - | 1 | - | - | - |
| 5HT2A (h) | - | - | - | - | 27 | 12 | - | 22 |
| 5HT3 (h) | 7 | 16 | - | 8 | 29 | 19 | 18 | 17 |
| 5HT5A (h) | 7 | 25 | 13 | - | 84 | 31 | 23 | 42 |
| 5HT2B (h) | - | - | 17.5 | - | 43 | - | 1 | 106 |
| Ca2+ Channel | 2 | 3 | - | - | 32 | 21 | - | - |
| Na+ Channel | 20 | - | - | - | 45 | 3 | - | 87 |
| CI Channel | 26 | 24 | 16 | 2 | 87 | 66 | 45 | 84 |
| K Channel | - | - | - | - | 9 | - | 17 | 2 |
| SK+ Ca | - | - | 1 | - | 1 | - | 5 | 2 |
| SST | - | - | - | - | 6 | 1 | - | - |
| V1 a (h) | - | 10 | - | - | 19 | 10 | - | 12 |
| VIP 1 (h) | 3 | - | - | - | - | - | - | - |
| ɣ1 (h) | 5 | - | - | 16 | 5 | - | - | - |
| ɣ2 (h) | - | - | - | 10 | - | 10 | 5 | 15 |
